# Sterile Insect Technique: Successful Suppression of an *Aedes aegypti* Field Population in Cuba

**DOI:** 10.3390/insects12050469

**Published:** 2021-05-18

**Authors:** René Gato, Zulema Menéndez, Enrique Prieto, Rafael Argilés, Misladys Rodríguez, Waldemar Baldoquín, Yisel Hernández, Dennis Pérez, Jorge Anaya, Ilario Fuentes, Claudia Lorenzo, Keren González, Yudaisi Campo, Jérémy Bouyer

**Affiliations:** 1 Instituto Pedro Kourí, Autopista Novia del Mediodia, La Lisa, La Habana 11400, Cuba; zulema@ipk.sld.cu (Z.M.); misladys@ipk.sld.cu (M.R.); wbaldoquin@infomed.sld.cu (W.B.); yhbarrios@ipk.sld.cu (Y.H.); dennis@ipk.sld.cu (D.P.); jorge@ipk.sld.cu (J.A.); ilanis@ipk.sld.cu (I.F.); claudiafcb@ipk.sld.cu (C.L.); rene@ipk.sld.cu (K.G.); kerenyudaisi@gmail.com (Y.C.); 2 Centro de Aplicaciones Tecnológicas y Desarrollo Nuclear, Calle 30 y 5ta ave. Miramar, La Habana 11300, Cuba; efprieto@ceaden.edu.cu; 3 Insect Pest Control Subprogramme, Joint FAO/IAEA Centre of Nuclear Techniques in Food and Agriculture, IAEA Vienna, Wagramer Strasse 5, 1400 Vienna, Austria; rargiles@hotmail.com (R.A.); jeremy.bouyer@cirad.fr (J.B.)

**Keywords:** autocidal control, irradiation, gamma radiation sterilization, vector control

## Abstract

**Simple Summary:**

The sterile insect technique (SIT) is a species-specific and environment-friendly method of insect control that relies on the release of large numbers of sterile insects. Mating released sterile males with wild females leads to a decrease in the reproductive potential and to the local suppression of the target population. There is increased interest in applying this approach to manage disease-transmitting mosquito populations. The main focus of this pilot trial was to assess the efficacy of the SIT for the suppression of *Aedes aegypti* populations. Two areas in Havana city, Cuba, were selected as control and release trial sites. The presence, density and fertility of the target wild population were monitored through a network of ovitraps. Approximately 1,270,000 irradiated *Ae. aegypti* males were released in the 50 ha target area over a period of 20 weeks. The released mosquitoes showed excellent mating competitiveness and induced high levels of sterility in the wild *Ae. aegypti* population. The target natural population was suppressed as reflected in the ovitrap index and in the mean number of eggs/trap values which dropped to zero by the last 3 weeks of the trial. We conclude that the released sterile male *Ae. aegypti* competed successfully and induced significant sterility in the local target *Ae. aegypti* population, resulting in suppression of the vector.

**Abstract:**

Dengue virus infections are a serious public health problem worldwide. *Aedes aegypti* is the primary vector of dengue in Cuba. As there is no vaccine or specific treatment, the control efforts are directed to the reduction of mosquito populations. The indiscriminate use of insecticides can lead to adverse effects on ecosystems, including human health. The sterile insect technique is a species-specific and environment-friendly method of insect population control based on the release of large numbers of sterile insects, ideally males only. The success of this technique for the sustainable management of agricultural pests has encouraged its evaluation for the population suppression of mosquito vector species. Here, we describe an open field trial to evaluate the effect of the release of irradiated male *Ae. aegypti* on a wild population. The pilot trial was carried out in a suburb of Havana and compared the mosquito population density before and after the intervention, in both untreated control and release areas. The wild population was monitored by an ovitrap network, recording frequency and density of eggs as well as their hatch rate. A significant amount of sterility was induced in the field population of the release area, as compared with the untreated control area. The ovitrap index and the mean number of eggs/trap declined dramatically after 12 and 5 weeks of releases, respectively. For the last 3 weeks, no eggs were collected in the treatment area, clearly indicating a significant suppression of the wild target population. We conclude that the sterile males released competed successfully and induced enough sterility to suppress the local *Ae. aegypti* population.

## 1. Introduction

The viruses that cause chikungunya, dengue fever and Zika pose a major threat to global public health. These arboviruses are transmitted by *Aedes aegypti* and *Aedes albopictus* mosquitoes, which are well-established in tropical and subtropical regions. The incidence of chikungunya, dengue and Zika has increased dramatically over the past 50 years due to expanding vector populations, increased global travel, human population growth, rapid and unplanned urbanization and climate change [1]. Urban environments with crowded human populations living in unhygienic conditions in intimate association with increased *Ae. aegypti* populations provide the ideal conditions for dengue transmission [2].

As there is no vaccine available to prevent dengue fever, the main efforts are directed to prevent the proliferation of mosquito populations. Most of the vector suppression programs rely on insecticide applications and the reduction of mosquito breeding sites [3]. However, these suppression tools have failed to reduce *Aedes* mosquito populations in an effective and sustainable way. Additionally, indiscriminate use of chemical insecticides has been associated with significant environmental problems as well as adverse effects on human health. Epidemiological evidence revealed the harmful effects of insecticide exposure, including serious and fatal consequences such as cancer [4]. Therefore, the additional use of innovative methods is being considered in many regions, as recommended by WHO [5]. 

The sterile insect technique (SIT) is an environment-friendly pest control method, with no harmful effects on human health [6]. The technique relies on the mass-rearing and release of radiation-sterilized male insects that will not produce viable offspring after mating with wild-type females [7]. If sufficient sterile males are released, most of the crosses will be sterile, and with time, the number of native insects decreases and the ratio of sterile to wild insects increases, resulting in suppression of the native population [3].

The technology has been successfully applied against insect pests of agricultural and veterinary importance in an area-wide integrated pest management approach (AW-IPM) [8,9,10,11]. For mosquito vectors of human diseases, the SIT has not yet reached the operational level. Pilot trials are ongoing to determine whether the SIT is an efficient method for the population suppression of mosquito vector species, including *Aedes spp*. [12,13,14]. 

The primary dengue vector worldwide is *Ae. aegypti*. This mosquito lives in close association with human beings, with a remarkable preference for blood-feeding on humans [15]. The larvae can develop in diverse water reservoirs that are associated with domestic activities [16]. These features make it an ideal vector for dengue virus transmission, especially in large urban areas where the human population density is high and artificial water containers are abundant [15,16].

Progressive assessment of the SIT from laboratory to large cages that was carried out prior to the present study has been valuable because it allowed for the systematic study of possible effects on mosquito survival and performance under increasing natural conditions [17,18].

The urgent need of alternative and environment-friendly approaches to suppress populations of mosquito vector species has enhanced the interest and support of public health decision makers for the SIT. The objective of this pilot trial was to evaluate the efficacy of the SIT to suppress a field population of *Ae. aegypti*, as a first step towards the development of an AW-IPM program with an SIT component against this major vector species.

## 2. Materials and Methods

### 2.1. Ae. aegypti Strain

The mosquito colony was initiated with eggs collected in ovitraps deployed in the study area in 2018. The colony was maintained under standard controlled conditions 28 ± 2 °C, 80 ± 10% RH and 8 h light, 16 h dark cycle.

### 2.2. Mass-Rearing

The adults for egg production were reared in 61 cm × 61 cm × 61 cm mesh-covered aluminum frame cages (BioQuip, Compton, CA, USA) at a density of one mosquito per cm^2^ of resting surface, and a 1:2 male to female ratio. A 50 cm × 50 cm gauze panel was suspended inside the cage to limit the flight of mosquitoes, and to provide more resting surface. Adult colonies were maintained for three gonotrophic cycles. Sterilized gauze strips soaked with 10% honey and dechlorinated water were suspended inside the cages for feeding/hydrating the adults and were replaced daily to prevent fungal growth. The honey-soaked strips were removed 12 h before and during blood-feeding. Defibrinated porcine blood in collagen casings (Fibran, Girona, Spain) was provided once a week for 4 h for female feeding. The blood casings (10 mL) were heated in a warm water bath at 38 °C, placed on top of the cages, and covered with warm rice-bags. Casings and bags were re-heated every 15 min. After blood-feeding, the water-soaked gauze strips were removed to prevent oviposition. Plastic trays (20 cm × 10 cm × 8 cm) with inner walls lined with filter paper strips were filled with 300 mL of dechlorinated water and placed inside the cages for oviposition and as a water source. The trays were placed inside the cages 2 days after blood-feeding to induce synchronous oviposition and were removed 24 h later. Eggs on the filter papers were allowed to mature in a wet environment for 3 days. The filter papers were dried in an air-conditioned room, and thereafter the eggs were gently brushed off and stored in plastic containers for up to 3 months. A larval rearing rack consisting of trays (100 cm × 60 cm × 3 cm) each filled with 4 L of deionized water was used to rear the immature stages [19] at a density of 2 larvae/mL. The larvae density was obtained by using egg quantity-weight regression curves as described by Zheng M. et al. [20]. Plastic flasks with the desired quantity of eggs for each tray, were filled with 100 mL of 36 °C de-oxygenated water and kept under vacuum for 10 min for synchronous hatching. The newborn larvae were left overnight in clean water without food to induce homogeneous development. First, instar larvae were transferred to the trays. The IAEA standard diet (50% tuna meal, 36% bovine liver powder, and 14% brewer’s yeast) was provided daily at the rate of 0.2, 0.4, 0.8 and 0.6 mg/larvae for larval instars I, II, III and IV, respectively. After the onset of pupation, the immatures were collected by tilting the trays in the rack at convenient intervals to achieve the desirable range of pupal age. The collected biological material was sorted using a Fay-Morlan apparatus (John W. Hock Co, Gainesville, FL, USA). The remaining larvae were returned to the rearing trays at the original density, and the female pupae discarded. Male pupae were dosed by volume in 10 mL plastic tubes graduated for approximately 500 individuals. Batches of 6000 male pupae were kept in 1 L flat tissue-culture flasks (Thermo Fisher, Waltham, MA, USA) filled with 250 mL of dechlorinated water until the optimal age for irradiation was reached. The flasks without lids were placed horizontally to provide wide water surface.

### 2.3. Sterilization and Packing

Mosquito pupae were irradiated in ^60^Co Isogamma LLCo irradiator (Izotop, Budapest, Hungary) as close to emergence as possible to reduce somatic damage, i.e., not before the age of 30 h. An irradiation dose of 80 Gy was applied with a dose rate of 8 kGy/h. The irradiation canisters consisted of cylindrical plastic tubes (120 mm height, 45 mm diameter) which are commonly used for adult tests with insecticide impregnated papers. The mesh in the lid allowed the drainage of the water, and the easy handling of the pupae. Three tubes each containing 6000 pupae without water were placed vertically in the irradiation chamber.

After irradiation, the pupae were returned to the culture-flasks for transport and emergence. Cardboard boxes of 15 cm × 15 cm × 60 cm were placed horizontally and used as adult containers. For air flow and mosquito release, square holes of 10 cm × 10 cm were cut in the two smaller sides of the box and covered with a fine mesh fixed with a rubber band. Additionally, one 3 cm diameter hole was cut in one of the 15 cm × 60 cm sides of the box. The neck of a culture-flask with 6000 irradiated male pupae was introduced through this hole. Emerged adult mosquitoes tended to escape from the light through the neck of the flask into the boxes for resting. After all adults had emerged, the flasks were removed, and the holes covered with a 50 mL plastic tube coated with honey-soaked filter paper. The adults were additionally provided with a 10% honey solution and dechlorinated water in soaked cotton pads of 15 cm × 20 cm × 1 cm that were placed inside the boxes.

### 2.4. Field Trial Design and Study Sites

**Figure 1:**
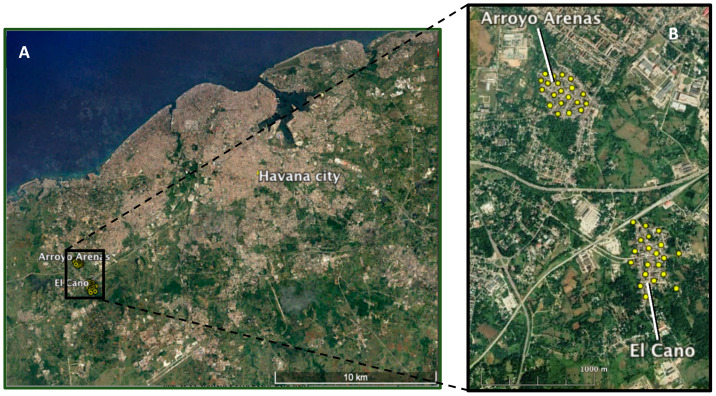
Satellite images showing the study sites. (**A**) Location southwest of Havana city. (**B**) Details from control (Arroyo Arenas) and the sterile male release (El Cano) sites; yellow dots indicate the ovitrap location. Image via Google Earth (21 January 2021).

The field trial was carried out in two comparable urban areas of the southwestern suburb of Havana city: Arroyo Arenas (23°02′47.1″ N 82°28′01.9″ W) and El Cano (23°01′59.8″ N 82°27′32.9″ W). The study sites were selected based on a predefined set of entomological, ecological, sociological and logistical criteria [21,22]. The two communities were isolated from each other and from the central metropolitan area of Havana by non-residential areas including forests, rivers, agricultural land, a railway and a national highway that were expected to minimize mosquito migration (Figure 1).

Non-marked sterile male mosquitoes were released in El Cano whereas Arroyo Arenas served as an untreated control area. Monitoring was carried out by collecting eggs from ovitraps that were counted under a stereomicroscope and hatched to assess the species identity and fertility. Trapping was initiated 8 weeks before the start of the releases to collect baseline data and ended 4 weeks after the last release. Ovitrap index, egg density and egg hatch rate were the outputs that were calculated each week. Ovitrap index is the proportion of ovitraps with at least one *Ae. aegypti* egg after 1 week in the field. Egg density was calculated as the mean number of eggs/trap.

According to the records of the national program of vector control, there is a history of continuous infestation by *Ae. aegypti* in both release and untreated control areas in recent years. *Ae. aegypti* uses indoor breeding sites including water storage containers and concrete water tanks, as well as artificial outdoor habitats. *Ae. albopictus* has rarely been encountered in natural breeding sites in the peri-urban areas.

The socioeconomic characteristics are similar between release and untreated control areas, with highly diverse occupational profiles and households with a modest standard of living. The typical houses are relatively small—one floor, two or three bedrooms and with a courtyard in the back, with running water, electricity, sewerage and regular rubbish collection.

The release and untreated control areas are linearly separated by 1200 m. El Cano has a surface of 50.2 ha, 3805 inhabitants and 906 houses distributed in 20 blocks, while Arroyo Arenas has 48 ha, 3726 inhabitants and 890 houses in 23 blocks.

### 2.5. Mosquitoes Releases

The release parameters were frequency, location and release rate that were set up based on the mosquitoes’ average life expectancy, flight range, and wild male abundance, respectively, as estimated by a mark-release-recapture trial. The release rate was initially restricted by production capacity and approximately 40,000 males were initially released for 3 consecutive weeks. When production capacity increased, the release rate was increased to 50,000, 60,000, 70,000 and 80,000 sterile males per week for 3, 5, 2 and 5 weeks, respectively. Due the low wild mosquito population density in the last 3 weeks, the release rate was reduced to 60,000 and 50,000 males per week for 1 and 2 weeks, respectively. The releases were initiated on 5 April 2020 (epidemiological week 15), corresponding to the beginning of summer, when wild mosquito populations tend to increase. The last sterile males were released on 29 August 2020 (epidemiological week 35). Cuba has two distinct seasons, a wet/hot one from late April to October and a dry/fresh one from November to early April.

The sterile mosquitoes were released shortly after sunrise (around 7:00 a.m.), when *Ae. aegypti* have a peak of activity [23], and the temperature and humidity are usually favorable (temperature ranged from 22.1 to 26.4 °C, and humidity ranged from 72 to 93%). Sterile non-marked males were released as 3-day-old adults by opening the lid of the boxes from a vehicle moving slowly (10–20 km/h) throughout the release area.

### 2.6. Monitoring System

The wild mosquito populations were monitored with a network of ovitraps deployed in both the release site and the untreated control area at a density of one ovitrap per block. The ovitrap consisted of a black 300 mL plastic cup lined with filter paper. Ovitraps were filled with tap water on site. The filter papers were collected weekly and transferred to the laboratory in plastic boxes. The eggs were allowed to mature under wet conditions for three days, then dried, counted and classified as field-hatched or non-hatched eggs. Papers with non-hatched eggs were immersed in hatching containers made from pipette-tip boxes (transparent, hinged lid), half-filled with dechlorinated water and tuna meal as food. The containers were checked daily; immatures were counted as third instar larvae and allowed to develop to adulthood. Adults were freeze-killed and morphologically classified at species level.

### 2.7. Estimation of Parameters for SIT-releases Settings

A mark-release-recapture trial was carried out 2 weeks before the start of the weekly releases. For the marking procedure, boxes with 2-day-old adult sterile males were individually placed in a fridge at 4 °C for 15 min. Immobilized mosquitoes were transferred in batches of around 3000 specimens to 1 L plastic containers containing 12.5 mg of fluorescent powder (DayGlo^®^ Color Corp., Cleveland, OH, USA). The containers were gently rotated for 10 s to achieve the contact of every mosquito with the powder [24]. Marked mosquitoes were transferred into 30 cm × 30 cm × 30 cm metallic frame cages (BioQuip, USA). The top side of these cages could be opened easily for releasing the sterile insects in the field. Mosquitoes were provided with water and honey. About 10,000 yellow-marked sterile males were released by ground from a single point in the center of the community “El Cano”.

The adult mosquitoes were monitored with 21 BG-Sentinel traps baited with BG-lures (Biogents, Regensburg, Germany) that were deployed in concentric rings of 50, 100, 150, 200, 250, 300 and 400 m, with each ring having 3, 3, 3, 4, 2, 1 and 5 traps, respectively. The adult traps were checked daily for 2 weeks and the sampled mosquitoes were transferred to the laboratory in plastic containers to prevent their crushing. The mosquitoes were killed at −20 °C and identified morphologically by species and sex under a stereoscope. Males were also classified as wild or marked under ultraviolet light.

The density of the wild male population was estimated using the Lincoln index [25]. The probability of sterile male daily survival (PDS) was estimated by fitting the exponential model to log-transformed data for recaptured males against the day of collection. The antilogarithm of the slope of the regression line gives an estimate of PDS. The average life expectancy of sterile males was calculated from the PDS as 1/–log_e_ PDS [26]. The flight behavior of released males was assessed as mean distance travelled and flight range [27].

### 2.8. Ethics Statement

The open field mosquito releases were approved by the government, the national health authorities and the regulatory agency for biological safety. All the activities of the national program of surveillance and vector control remained under normal operation in both release and untreated control areas. There were no mosquito-borne disease outbreaks reported during this study.

### 2.9. Social Issues

A community communication campaign was encouraged by family doctors and social leaders from their own study sites, who highlighted the safety of the release of sterile male mosquitoes. In order to avoid an additional intervention, no active community participation in mosquito control activities was promoted.

### 2.10. Data Analysis

The data were statistically analyzed using R Software version 3.5.2 (R Development Core Team, Vienna, Austria) [28].

The frequency (ovitrap index) and density of eggs of *Ae. aegypti* per trap (eggs/trap) were averaged per time unit (week). The percentage of induced egg sterility was calculated by Equation (1), proposed by Bellini et al. [29,30].

S = 1 − ((Eh_I_/E_I_) (E_C_/Eh_C_)) (1)

where S is the percent egg sterility, Eh is the mean number of hatched egg per ovitrap per week, E is the mean number of eggs per trap per week, I is the intervention area and C is the control area.

The competitiveness index as defined by Fried [31] was calculated using the egg hatch rate from the untreated control and release areas (Equation (2)).

Fried Index = (W/S) ((P_W_ − P_S_)/(P_S_ − P_RS_)) (2)

where W and S are the number of wild and sterile males, respectively, P_W_ is the percentage egg hatch in the untreated control area, P_S_ is the percentage egg hatch in the release area and the assumption of residual fertility of sterile males (P_RS_) is 3%.

The effect of the releases was assessed by an interrupted time series analysis with a control group. A common trend model was used [32,33]. The explanatory variable (*x_t_*) was the egg density. The model allowed comparison between pre- and post-intervention data as well as intervention vs. control. We implemented a linear estimating equation regression model, as follows:

(3) $$d_{t}=y_{I_{t}}-y_{C_{t}}=\alpha_{d}+\beta_{1}x_{1t}+\beta_{2}x_{2t}\left(t-T\right)+\epsilon_{dt}$$

where $\alpha_{d}=\alpha_{I}-\alpha_{C}\;$and $\epsilon_{dt}=\epsilon_{It}-\epsilon_{Ct}$. Thus, the intervention effect, β can be estimated by performing a regression where the difference, $d_{t}$, is the outcome and $x_{t}$ is the explanatory variable. ϵ is an error term, t is the time unit, T is the time elapsed since the start of the study, I is the intervention, C is the control, $\beta_{1}$ is the effect in the roll out period, $\beta_{2}$ represents the change in the intervention effect for each unit increase in time, $x_{1t}$ is an indicator for the roll out period and $x_{2t}$ is an indicator for de intervention period. Confidence intervals were calculated using Newey–West standard errors.

## 3. Results

Similar values of the ovitrap index, mean number of eggs/trap and hatch index were observed in the El Cano and Arroyo Arenas sites prior to the start of releases (*p* > 0.05).

The data of the mark-release-recapture trial showed that the marked sterile males dispersed an average of 77.3 m. The flight range varied between 43.2 m (50%) and 110.5 m (90%), and the average lifespan was 3.76 days. The relative abundance of the wild male population during this trial was estimated at 130.3 males/ha.

Based on these results, it was decided to release a minimum of 40,000 male mosquitoes/week at release points separated by 200 m and a release frequency of two times per week.

By the time the weekly releases were initiated, the wild mosquito populations were low because of the seasonal fluctuation. Approximately 1,270,000 irradiated *Ae. aegypti* males were released for 21 weeks in the pilot trial site. The release of 40,000 sterile males/week for 3 consecutive weeks represented an initial sterile to wild male ratio of 6.4:1. However, this ratio increased as the releases progressed as a result of a substantial increase in the release rate from 800 to 1600 sterile males/ha/week.

**Figure 2:**
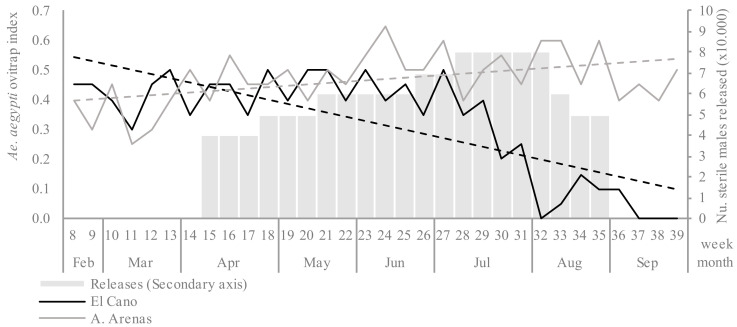
Ovitrap index of *Aedes aegypti* (solid lines, left Y axis) between epidemiological weeks 8 and 39, 2020, in the release (El Cano) and untreated control (Arroyo Arenas) areas, and the linear trend (dashed lines). The gray bars indicate the number of sterile males released (weeks 15–35) in El Cano (right Y axis).

The mean ovitrap index in the first 8 weeks (baseline) was similar in the El Cano and Arroyo Arenas sites (0.41 and 0.37, respectively) (*p* > 0.05). In the untreated control area, the ovitrap index increased throughout the trial period as expected by season fluctuation, with a mean of 0.49 in the last 3 months (Figure 2). In the release area, the ovitrap index initially fluctuated under 0.5 after the start of the releases, but there was a consistent decline after epidemiological week 29, reaching zero positive ovitraps for 3 weeks at the end of the trial.

**Figure 3:**
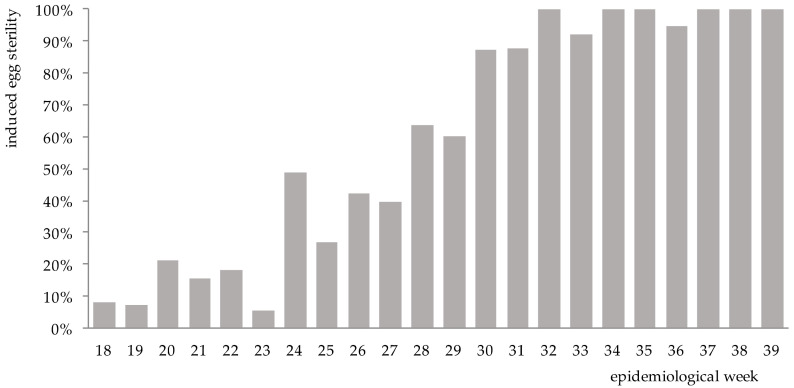
Weekly mean induced egg sterility (%) in the release site El Cano between epidemiological weeks 18 and 39, 2020.

The increase in sterility of *Ae. aegypti* eggs became evident by epidemiological week 20 (5 weeks post-release) in the release area relative to untreated control (Figure 3). In subsequent weeks, the induced sterility increased notably, and no viable eggs were collected for up to 6 weeks.

**Figure 4:**
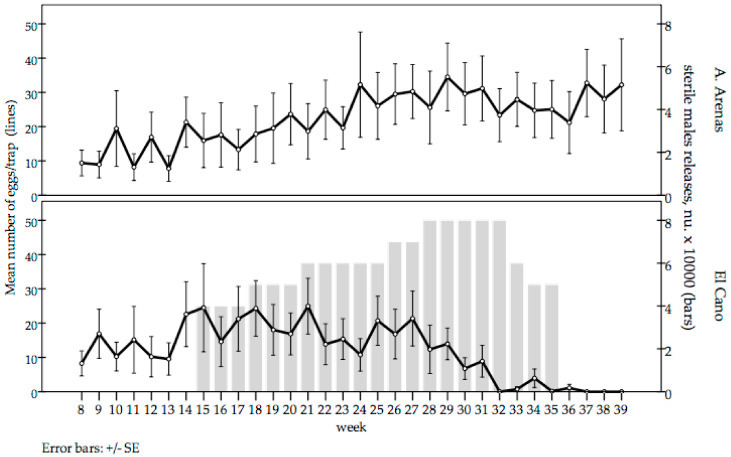
*Aedes aegypti* mean number of eggs/trap in the release (El Cano) and untreated control (Arroyo Arenas) areas between epidemiological weeks 8 and 39, 2020 (left Y axis). The gray bars indicate the number of sterile males released (weeks 15–35) in El Cano (right Y axis).

The mean number of eggs/trap were similar during the pre-release period in El Cano and Arroyo Arenas sites. There was a significant reduction in the mean number of eggs collected per trap in El Cano after 5 weeks of releases. The mean number of eggs/trap in El Cano became zero after 17 weeks of releases. Thereafter, a mean of five eggs per trap were collected for 4 weeks, but no eggs were collected during the last 3 weeks, indicating a significant reduction of the wild population density. In contrast, the mean number of eggs/trap in the untreated control area tended to increase during the trial period. During epidemiological weeks 37 to 39, the values of El Cano remained null, whereas in the untreated control area, the mean collections were 32.75, 28.05 and 32.2 eggs/trap, respectively (Figure 4).

The difference in egg density was evidenced by the interrupted time series analysis (before and after the releases) and by comparing intervention-control time series (common trend model). The roll out period was from epidemiological week 15 to 19. The value of the outcome of the common trend model was 1.5.

The competitiveness index [31] during epidemiological weeks 17 to 20 was 0.56, assuming a residual fertility of 3% in released mosquitoes (Table 1).

## 4. Discussion

This pilot study demonstrated the effectiveness of the SIT to suppress an *Ae. aegypti* population under field conditions in Cuba. The release of sterile male mosquitoes started at the end of winter, when the population density is traditionally low. Thus, it was not considered necessary to suppress the mosquito population with another control method before applying the SIT.

The density of 130.3 wild males/ha found in the mark-release-recapture trial is considered very high in Cuba, according to the standards of the national program of vector control. However, these standards are based on human bait, a subjective method with poor accuracy.

The selection of an appropriate pilot study site is critical for obtaining solid data [21,34]. Despite the logistical complexity associated with metropolitan areas, two urban neighborhoods belonging to Havana were selected, as *Ae. aegypti* is predominantly an urban species [35]. El Cano and Arroyo Arenas are partially ecologically isolated from each other and from surrounding neighborhoods. The size, shape, architecture and social structure were relatively similar between both sites. These areas also had a history of presence of *Ae. aegypti* for many years, as was corroborated in this study by monitoring the mean number of eggs/trap during the pre-release phase.

Ovitraps represent a simple and effective method for monitoring *Aedes* mosquito populations. Ovitrap surveillance data correlated well with other calculated indices that were used to estimate seasonal population dynamics of *Ae. albopictus* in Italy [23]. The use of adult mosquito traps is labor intensive, as pointed out by Reiter [36], and inconvenient. The BG-Sentinel traps have to be installed indoors for safety. However, the typical houses in the study area are small and the BG-lure releases a strong smell. In addition, the deployment of a network of traps requires daily visits for collecting the catches for a long period. In this study, the sterile males were not marked to avoid damage, which could compromise their competitiveness. Therefore, the adult wild population was not monitored directly, the sterile:wild ratio could not be estimated and, consequently, the competitiveness could not be determined during the entire trial.

The initial sterile to wild male ratio was sub-optimal due to the low mass-rearing capacity. Nevertheless, a previous study carried out in our laboratory showed that the weekly release of chemo-sterilized males at a 5:1 ratio with the fertile males was sufficient to eliminate a caged population of *Ae. aegypti* within 15 weeks [18]. Certainly, the trials on the effect of sterile mosquitoes on the suppression of mosquito-caged populations should be interpreted with caution; however, they do provide some indication of the efficacy of the technique, as part of the progressive, stepwise evaluation of sterile mosquitoes [37]. We assumed higher overflooding ratios after epidemiological week 17, as the number of released sterile males was noticeably increased from 50,000 to 80,000 males per week once the mass-rearing was improved, whereas we observed a reduction in the number of eggs collected per trap from the field.

In a similar way, Zheng et al. aimed for a sterile to wild male *Ae. albopictus* ratio of 5:1 at the beginning of their pilot study in China. The released mosquitoes (irradiated plus *Wolbachia* infected) at this ratio appeared to be able to induce high degrees of sterility [38].

In our facility, we achieved a significant enhancement in efficiency of the mass-rearing by epidemiological week 28, resulting in a production of over 120,000 males/week. However, the releases were intentionally restricted to 1600 male mosquitoes per ha, to prioritize the assessment of a realistic overflooding ratio for further extended scenarios.

The existence of *Ae. albopictus* in the pilot site was a challenge for monitoring since their eggs are morphologically indistinguishable from those of *Ae. aegypti*. It was managed by allowing the mosquitoes to reach adulthood for identification. However, it is a time and space-consuming task, and the need of a different approach for wider studies is clear.

The Fried competitiveness index was calculated for epidemiological weeks 17 to 20 based on an accurate estimation of the wild population density. Further estimations were not feasible as the sterile males were not marked. The Fried index of 0.56 reflected the excellent ability of male mosquitoes to induce sterility in the wild population. This was higher than the competitiveness index value of 0.26 (95% confidence interval, 0.05 to 0.72) that was obtained during a field study with *Ae. aegypti* in Brazil [39]. A recent study in Mexico reported a competitiveness between 0.09 and 0.46 for 70 Gy-irradiated *Ae. aegypti* males, but the experiments were carried out in field cages [40]. A high Fried index of 0.86 was also found when the sterile:fertile male ratio was 5:1 in another cage study with *Ae. aegypti* in Thailand [41]. Results from mating competitiveness trials in cages have generally been found to underestimate the performance of irradiated mosquitoes in the field [42]. In the Cayman Islands (2012) and Brazil (2015), the genetically modified OX513a strain from Oxitec showed competitiveness values of 0.059 (0.011–0.210) and 0.031 (0.025–0.036), respectively, during field trials, much lower than the values reported for irradiated *Ae. aegypti* males in the SIT trials [43,44]. In Italy, the field competitiveness of irradiated *Ae. albopictus* was estimated through the level of induced sterility each week. The authors reported a strong negative correlation between the field competitiveness of the *Ae. albopictus* males released and the ratio of sterile to wild males [29].

In our study, the wild population suppression in the release area was evaluated by monitoring the presence of mosquito eggs, their density and hatch rate in the ovitrap network. The interrupted time series analysis indicated that the reduction in the mean number of eggs/trap after the epidemiological week 19 was caused by the SIT intervention. The common trend model displayed a reliable confirmation of the effect of the intervention by comparing time series in treated versus control area. Assuming the common trend model, we eliminate the effect of the unobserved confounders (the trend) by subtracting the control series $y_{C_{t}}$ from the intervention series $y_{I_{t}}$. The outcome of the model suggests that the intervention reduced the average of mosquito eggs per trap by 1.5 (95% CI: −1.76 −1.39) for each unit (week) increase in time. In a previous laboratory study of caged *Ae. aegypti* population, we described a finite rate of natural increase of 2.92, and an intrinsic rate of natural increase of 1.07 [17]. Therefore, the common trend model seems to be plausible. The model also evidenced a time lag between the beginning of the intervention and the observed effects on indicators. This roll-out period ranged from 5 weeks for mean number of eggs/trap to 12 weeks for the ovitrap index.

As far as we know, this is the first study that reports the suppression of a field population of *Ae. aegypti* by applying SIT alone. However, similar studies have been conducted for the suppression of *Ae. aegypti* populations by other genetic control methods. For example, using transgenic mosquitoes, population suppression of *Ae. aegypti* was reported in the Cayman Islands (80%), Brazil (85%) and Panama (93%) [43,44,45]. Recently, Mains et al. achieved a significant reduction in the number of *Ae. aegypti* in an urban neighborhood in a metropolitan area of Miami, USA, by releasing *Wolbachia*-infected males [46]. In this case, the cytoplasmic incompatibility was used as sterilization method [47]. In Thailand, Kittayapong et al. demonstrated the reduction of natural populations of *Ae. aegypti* in a semi-rural village by combining SIT with *Wolbachia*-induced incompatibility (SIT/IIT) [41].

Bellini et al. reported the suppression of a target *Ae. albopictus* population in field trials in Italy [30,48]. Successful suppression of target populations of *Ae. albopictus* was also reported by Zheng et al. during the largest open field trials ever conducted using the combined SIT/IIT approach over the residential areas of two islands in China [38].

## 5. Conclusions

We conclude that the irradiated males released during this SIT field trial in Cuba successfully competed with wild males and induced sufficient sterility to suppress the local *Ae. aegypti* population. The findings from this study provide optimism to initiate larger scale trials directed to estimate the impact of the SIT on epidemiological outcomes. The area-wide sustained release of irradiated males is a promising tool to be integrated with existing control methods for the management of the diseases transmitted by *Ae. aegypti*.

## Figures and Tables

**Table 1:** Hatch rate per week of *Aedes aegypti* field collected eggs in release (El Cano) and untreated control (Arroyo Arenas) areas and Fried index of sterile males released, epidemiological weeks 17–20.

Area	Hatch Rate Per Week		Sterile:Wild Ratio	Fried Index
	Mean (%)	SD		
Release	79.77	7.30	6.43:1	0.56
Untreated control	86.47	4.13		

## Data Availability

The data presented in this study are available on request from the corresponding author.
